# Inhibitory Effect of LGS and ODE Isolated from the Twigs of *Syringa oblata* subsp. *dilatata* on RANKL-Induced Osteoclastogenesis in Macrophage Cells

**DOI:** 10.3390/molecules26061779

**Published:** 2021-03-22

**Authors:** Ga-Ram Kim, Eun-Nam Kim, Kyoung Jin Park, Ki Hyun Kim, Gil-Saeng Jeong

**Affiliations:** 1 College of Pharmacy, Keimyung University, 1095 Dalgubeol-daero, Daegu 42601, Korea; o930302@naver.com (G.-R.K.); enkimpharm@gmail.com (E.-N.K.); 2 School of Pharmacy, Sungkyunkwan University, Suwon 16419, Korea; pkjin6515@skku.edu

**Keywords:** *Syringa oblata* subsp. *dilatata*, osteoclastogenesis, ligustroside, oleoside dimethylester

## Abstract

Osteoblasts and osteoclasts play a pivotal role in maintaining bone homeostasis, of which excessive bone resorption by osteoclasts can cause osteoporosis and various bone diseases. However, current osteoporosis treatments have many side effects, and research on new treatments that can replace these treatments is ongoing. Therefore, in this study, the roles of ligustroside (LGS) and oleoside dimethylester (ODE), a natural product-derived compound isolated from *Syringa oblata* subsp. *dilatata* as a novel, natural product-derived osteoporosis treatments were investigated. In the results of this study, LGS and ODE inhibited the differentiation of receptor activator of nuclear factor kappa-Β ligand (RANKL)-induced RAW264.7 cells into osteoclasts without cytotoxicity, and down-regulated the activity of TRAP, a specific biomarker of osteoclasts. In addition, it inhibited bone resorption and actin ring formation, which are important functions and features of osteoclasts. Also, the effects of LGS and ODE on the mitogen-activated protein kinase (MAPK) and nuclear factor kappa-light-chain-enhancer of activated B (NF-κB) and phosphoinositide 3-kinases (PI3K)/ protein kinase B (Akt)/mechanistic target of rapamycin (mTOR) signaling pathways that play important roles in osteoclast differentiation were evaluated. In the results, LGS and ODE downregulated the phosphorylation of RANKL-induced MAPK and PI3K/Akt/mTOR proteins in a concentration-dependent manner, translocation of NF-κB into the nucleus was inhibited. As a result, the compounds LGS and ODE isolated from *S. oblate* subsp. *dilatata* effectively regulated the differentiation of RANKL-induced osteoclasts and inhibited the phosphorylation of signaling pathways that play a pivotal role in osteoclast differentiation. Therefore, these results suggest the possibility of LGS and ODE as new natural product treatments for bone diseases caused by excessive osteoclasts.

## 1. Introduction

Osteoporosis affects men as well as postmenopausal women, and most fractures occur mainly in the hip, spine, and wrist [1]. Fractures due to osteoporosis not only adversely affect the quality of life but are also associated with increased mortality and increased medical costs [2]. The world population is steadily increasing so the social and economic burden of osteoporosis is also enhancing due to aging; it is a major public health problem throughout the world by the incidence of osteoporosis is increment yearly with the aging [3,4].

Bone is a dynamic tissue that plays important roles in the body such as physical support functions, organ protection functions, and regulation of mineral homeostasis [5]. The balance of osteoclasts that resorption bone and osteoblasts that produce bone plays a very important role in bone health and bone is constantly destroyed and produced and maintains homeostasis [6,7]. Among them, osteoclasts are multinuclear giant cells that are involved in bone resorption [8]. These osteoclasts are known to induce diseases such as osteoporosis by absorbing the bone surface, and mature osteoclasts adsorb to the bone by secreting tartrate-resistant acid phosphatase (TRAP) related to bone resorption and initiate bone resorption [9,10]. An important role in the treatment of osteoporosis is directly related to the recovery of bone cells, and osteoporosis is mainly caused by excessive bone resorption due to excessive osteoclast formation [11]. Therefore, suppressing the production of excessive osteoclasts is one of the important treatment strategies for osteoporosis treatment.

The receptor activator of nuclear factor kappa-Β ligand (RANKL)/receptor activator of nuclear factor-kappa B (RANK) pathway plays a critical role in bone remodeling and a pathway associated with osteoclast and osteoblast, and when progenitor cells such as macrophage are stimulated with RANKL and M-CSF, differentiation into osteoclasts occurs [12]. RANKL activates RANK to stimulate differentiation against precursors of osteoclasts, attach osteoclasts to bone tissue, and maintain and activate them RANKL is mainly expressed in osteoblasts and binds to RANK receptors on the surface of pre-osteoclasts to induce osteoclast differentiation and signal transduction pathway activation [13,14,15]. In many previous studies, it has been reported that binding of RANKL to the receptor RANK promotes the mobilization of molecules such as tumor necrosis factor receptor (TNFR)-associated factor 6 (TRAF 6), thereby activating mature osteoclasts through the activation of transcription factors such as nuclear factor activated T cells 1 (NFATc1), c-Fos, and nuclear factor kappa-light-chain-enhancer of activated B (NF-κB) [16,17].

*Syringa oblata* subsp. *dilatata* (Nakai) P.S. Green & M.C. Chang (Oleaceae) is well-known as Korean early lilac [18], and it features showy panicles of fragrant lilac purple flowers. In China, it has been used as a folk medicine for the treatment of vomiting, diarrhea rheumatoid arthritis and hernia [19]. *S. olbata* subsp. *dilatata* is reported to contain secoiridoid glucosides as major compounds, some of which showed antioxidant activity [20]. In our recent chemical investigation of *S. olbata* subsp. *dilatata*, we identified 15 secoiridoid glucosides, including two new secoiridoid glucosides (dilatiosides A and B), ligustroside (LGS) and oleoside dimethylester (ODE), which were evaluated for their effects on the induction of nerve growth factor (NGF) secretion in a C6 rat glioma cell line and their cytotoxicity against several cancer cell lines [18]. However, LGS and ODE were not active, although the compounds, LGS and ODE, have been known to show anti-inflammatory and antiviral activities in the previous study [21].

In this study, the phytochemical analysis of *S. olbata* subsp. *dilatata* led to the further isolation of LGS (7 mg) and ODE (130 mg). Until now, the effects of *S. olbata* subsp. *dilatata* and its components on osteoporosis have not been reported. In addition, the biological activities of the compounds, LGS and ODE have not been explored in terms of osteoclast formation. Therefore, in this study, the effects of LGS and ODE on osteoclast differentiation in RAW264.7 macrophages induced osteoclast differentiation with RANKL were investigated.

## 2. Result

### 2.1. Effect of LGS and ODE on Cytotoxicity in RAW264.7 Cells

**Figure 1:**
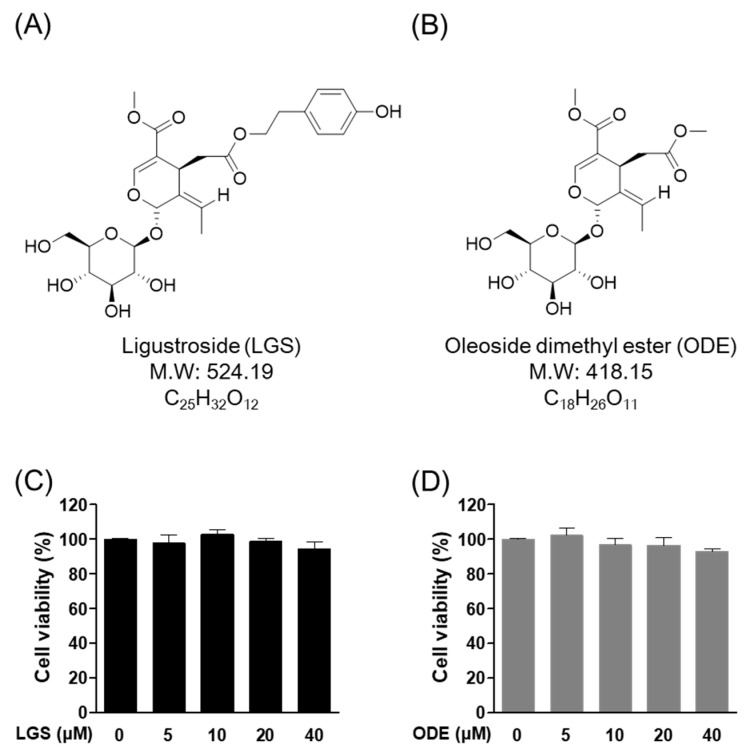
Effect of ligustroside (LGS) (**A**) and oleoside dimethylester (ODE) (**B**) on cytotoxicity in RAW 264.7 cells. The RAW 264.7 cells were treated with various concentrations of LGS (**C**) and ODE (**D**) (5, 10, 20, 40 μM) for 7 days and cell viability was determined by MTT assay. Mean value of three experiments ± S.D. was presented.

The 3-(4,5-Dimethylthiazol-2-yl)-2,5-Diphenyltetrazolium Bromide (MTT) experiment was performed to confirm the cytotoxicity of LGS (Figure 1A) and ODE (Figure 1B) (Table 1) in RAW 264.7 cells. Each of the indicated concentrations (5 to 40 μM) of LGS and ODE was treated in RAW 264.7 cells for 7 days in the same manner as the osteoclast differentiation conditions. As a result of evaluating the cell viability through MTT analysis 7 days after LGS (Figure 1C) and ODE (Figure 1D) treatment, no significant toxicity within the standard deviation was found in the concentrations of 5, 10, 20, and 40 μM. Therefore, subsequent experiments were conducted in the concentration range of 5 to 40 μM for both LGS and ODE.

### 2.2. Inhibitory Effect of LGS and ODE on Actin Ring Structure and TRAP Activity during RANKL-Induced Osteoclast Formation

**Figure 2:**
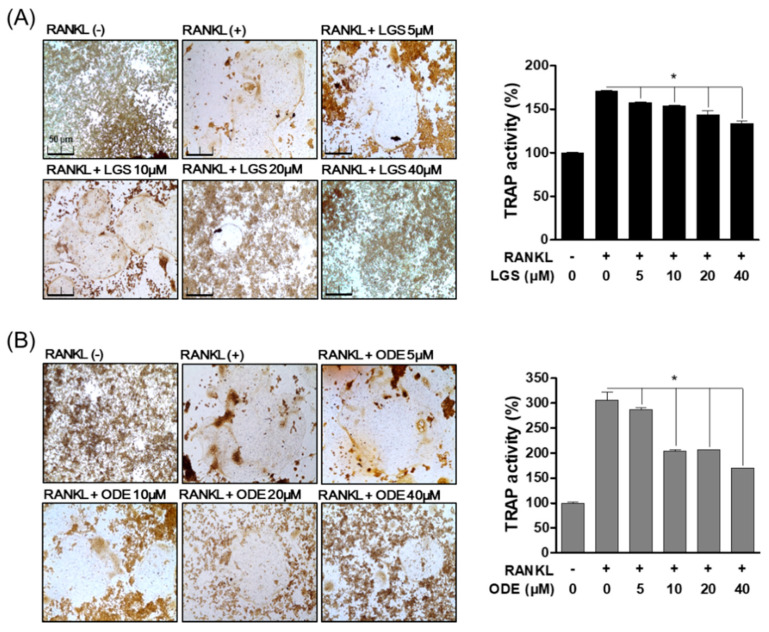
Inhibitory effect of LGS and ODE on osteoclast differentiation. RAW 264.7 cells were cultured for 7 days with or without RANKL (50 ng/mL) in the indicated concentration of LGS (**A**) and ODE (**B**). Cells were fixed in 4% formaldehyde and stained for TRAP and measured TRAP activity. Mean value of three experiments ± S.D. was presented. * *p* < 0.05 versus the RANKL-treated group.

**Figure 3:**
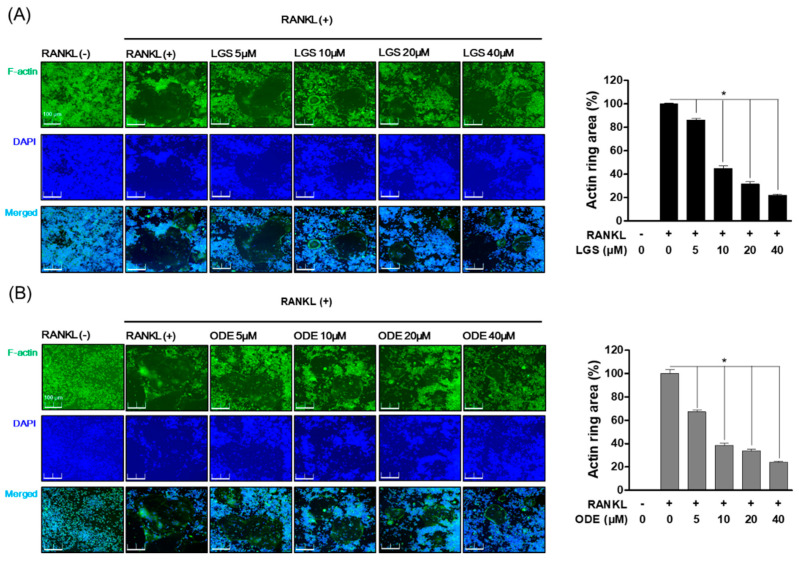
Inhibitory effects of LGS and ODE on the actin ring structure formation during osteoclast formation. RAW 264.7 cells were cultured in 24-well plates (5 × 10^3^ cell/well) for 7 days in the presence or absence of LGS (**A**) and ODE (**B**) with RANKL (50 ng/mL). Cells were fixed in 0.1% Triton X-100, after then stained with Alexa 488 and 4′,6-diamidino-2-phenylindole (DAPI). The mean value of three experiments ± S.D. was presented. * *p* < 0.05 versus the RANKL-treated group.

To investigate the effect of LGS and ODE on osteoclast differentiation, we evaluated the activity of tartrate-resistant acid phosphatase (TRAP), known as the specific biomarker of osteoclasts, and the effect of LGS and ODE was evaluated on the formation of the actin ring structure, a structural feature of osteoclasts. In RAW 264.7 cells, LGS and ODE were treated with RANKL for 7 days to evaluate the effects of LGS and ODE in an in vitro model of osteoclast differentiation. As a result, compared to the control group treated with only RANKL, LGS and ODE inhibited the formation and activity of TRAP (Figure 2A,B), a specific biomarker of osteoclasts, in a dose-dependent manner at concentrations of 5, 10, 20, and 40 μM. In addition, LGS and ODE suppressed the formation area of the actin ring formed for bone absorption in a concentration-dependent manner, and at the same time, suppressed the number of nuclei of osteoclasts, which are giant multinuclear cells (Figure 3A,B). These results suggest that LGS and ODE effectively regulate the differentiation of osteoclasts induced excessively by RANKL, thereby suggesting the potential as an osteoclast differentiation inhibitor.

### 2.3. LGS and ODE Inhibit Bone Resorption by RANKL-Induced Osteoclasts

**Figure 4:**
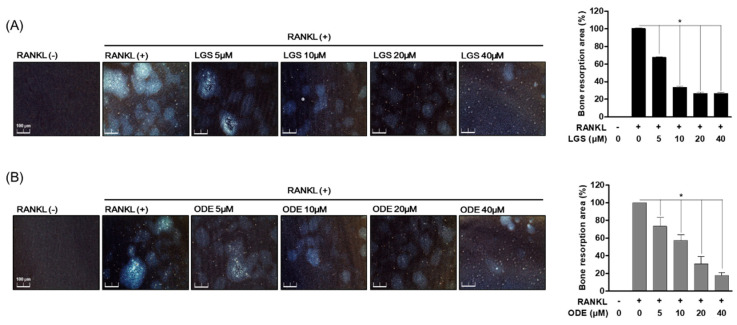
LGS and ODE inhibit bone resorption by RANKL-induced osteoclasts. RAW 264.7 cells (2 × 10^3^ cell/well) were cultured during 7 days treated or untreated with RANKL, ODE (**A**) and LGS (**B**) in an osteo-surface assay plate. Cells were removed and resorption pits were identified by light microscopy. Mean value of three experiments ± S.D. was presented. * *p* < 0.05 versus the RANKL-treated group.

LGS and ODE inhibited osteoclast differentiation-specific biomarkers and formation. Therefore, the effects of LGS and ODE were evaluated on bone resorption, one of the major functions of RANKL-induced osteoclasts. First, RAW 264.7 cells were simultaneously treated with RANKL (50 ng/mL), LGS, and ODE (5–40 μM) for 7 days and cultured in an osteo-surface assay well, and then the bone resorption area by differentiation of osteoclasts was compared with the only RANKL treatment group. After 7 days, the differentiated osteoclasts were removed, and as a result of evaluating the bone resorption area of the bone surface by the osteoclasts, it was confirmed that the group treated with LGS (Figure 4A) and ODE (Figure 4B), together with RANKL, reduced the bone resorption area compared to the group treated with only RANKL. The inhibitory effect of LGS and ODE on bone resorption by osteoclasts suggests the potential as a regulator of not only the differentiation and formation of osteoclasts, but also the major functions of osteoclasts.

### 2.4. LGS and ODE Inhibit RANKL-Induced Osteoclast Formation Master Transcription Factor and Osteoclast-Specific Gene Expression

**Figure 5:**
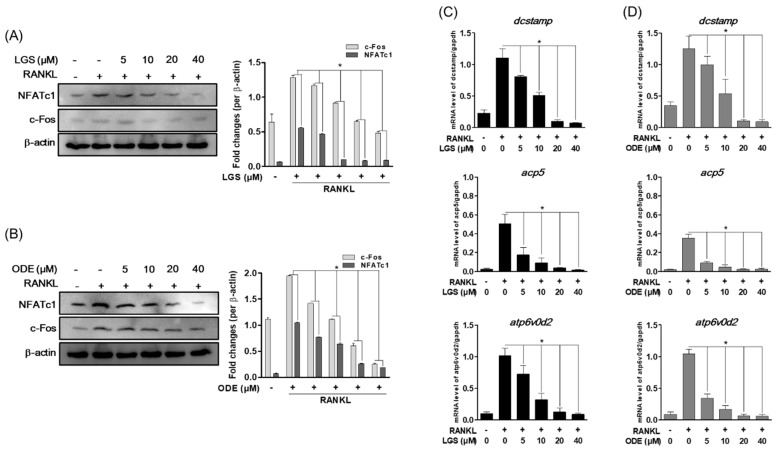
LGS and ODE inhibit RANKL-induced osteoclast formation master transcription factor and osteoclast-specific gene expression. RAW 264.7 cell were pretreated with or without LGS and ODE for 2 h and then treated with RANKL (50 ng/mL) for 24 h. Cellular proteins were extracted and identified c-Fos and NFATc1 dependent pathways through western blot analysis. Expression levels of c-Fos and NFATc1 were quantified by western blotting (**A**,**B**). LGS and ODE regulate RANKL-mediated osteoclast-specific gene expression. Osteoclast-specific gene expression was analyzed using real-time PCR and results were standardized to the expression of glyceraldehyde 3-phosphate dehydrogenase (GAPDH) (**C**,**D**). The mean value of three experiments ± S.D. was presented. * *p* < 0.05 versus the RANKL-treated group.

In the process of osteoclast differentiation and formation, master transcription factors, such as nuclear factor of activated T-cells (NFATc), 1 protein, and c-Fos act to induce the expression of osteoclast-specific genes. Therefore, the effect of LGS and ODE on the expression of these master transcription factors and osteoclast-specific genes was evaluated, such as dendritic cell-specific transmembrane protein (*DC-STAMP*), acid phosphatase 5 (*ACP5*), and ATPase H+ transporting V0 subunit D2 (*ATP6v0d2*) during RANKL-induced osteoclast differentiation. As a result, first, LGS (Figure 5A) and ODE (Figure 5B) suppressed the protein expression of the master transcription factors Nfatc1 and c-Fos induced by RANKL in a concentration-dependent manner. In addition, the mRNA levels of DC-STAMP, ACP5, and ATP6v0d2, which are osteoclast-specific genes upregulated by RANKL, were effectively suppressed (Figure 5C,D). These results suggest the possibility that LGS and ODE can exhibit the previously shown osteoclast differentiation and function inhibitory effects through the inhibition of the master transcription factor and the specific gene of osteoclasts.

### 2.5. LGS and ODE Inhibit the Pathways of MAPK and NF-ĸB in Osteoclastogenesis Induced by RANKL

**Figure 6:**
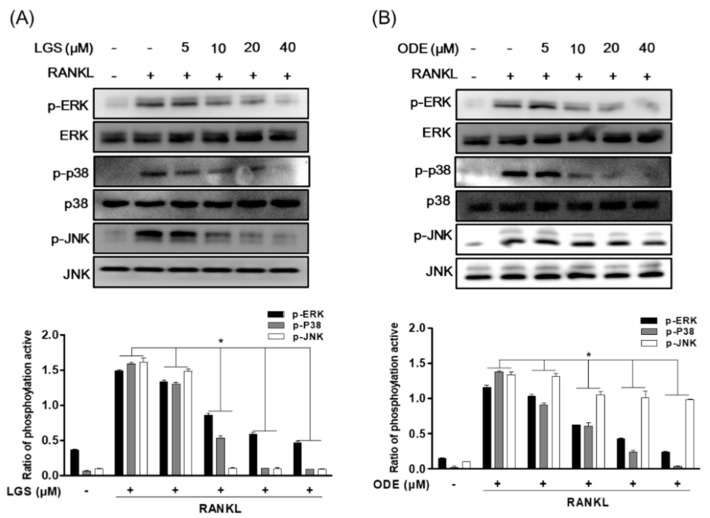
LGS and ODE inhibit the pathways of MAPK in osteoclastogenesis induced by RANKL. RAW 264.7 cells were pretreated with or without LGS (**A**) and ODE (**B**) for 2 h and treated with RANKL (50 ng/mL) for 30 min. Then, the whole protein was extracted and western blotting was performed. Quantitated signals are plotted. The gel images are representative of three independent experiments. Mean value of three experiments ± S.D. were presented. * *p* < 0.05 versus the RANKL-treated group.

MAPK and NF-κB activation induced in response to binding to RANKL and RANK are known to play a role in regulating several downstream signaling molecules, including the expression of osteoclast-specific genes, such as TRAP and OSCAR. Therefore, to investigate the mechanism by which LGS and ODE inhibit osteoclastogenesis, this study confirmed the effect of LGS and ODE on the activity of MAPK and IĸBα induced by RANKL. The signal transduction pathway was confirmed 30 min after RANKL treatment was performed on RAW 264.7 cells treated or untreated with LGS and ODE. As a result, when RANKL were treated together LGS (Figure 6A) or ODE (Figure 6B), phosphorylation of p38, JNK and ERK decreased dose-dependently.

**Figure 7:**
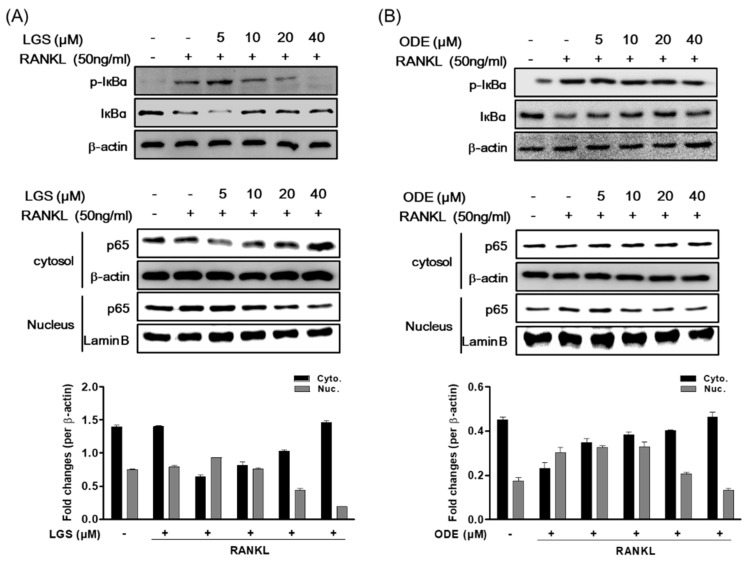
RAW 264.7 cells were pretreated with or without LGS (**A**) and ODE (**B**) for 2 h and then treated with RANKL (50 ng/mL) for 30 min. In order to confirm the expression of NF-κB, the cytoplasm and nucleus were separated according to the instructions to measure protein expression of IκBα, and NF-κB p65. Cell lysates were analyzed by Western blot. Quantitated signals are plotted. The gel images are representative of three independent experiments. The mean value of three experiments ± S.D. was presented.

In addition, LGS and ODE inhibited the phosphorylation of IĸBα in a dose-dependent manner. It was confirmed that the activity of NF-ĸB induced by RANKL was also inhibited by treating LGS and ODE. Translocation of p65, induced by LGS and ODE, was confirmed using a protein extract obtained by separating the cytoplasm and nucleus by western blotting (Figure 7A,B). When treated with LGS and ODE, p65 significantly inhibited nuclear translocation. From these results, it was confirmed that the osteoclast inhibitory effect of LGS and ODE was related to the inhibition of the signaling pathway activity of p38, JNK, ERK and IĸBα.

### 2.6. LGS and ODE Inhibit the Pathways of PI3K/Akt/mTOR in Osteoclastogenesis Induced by RANKL

**Figure 8:**
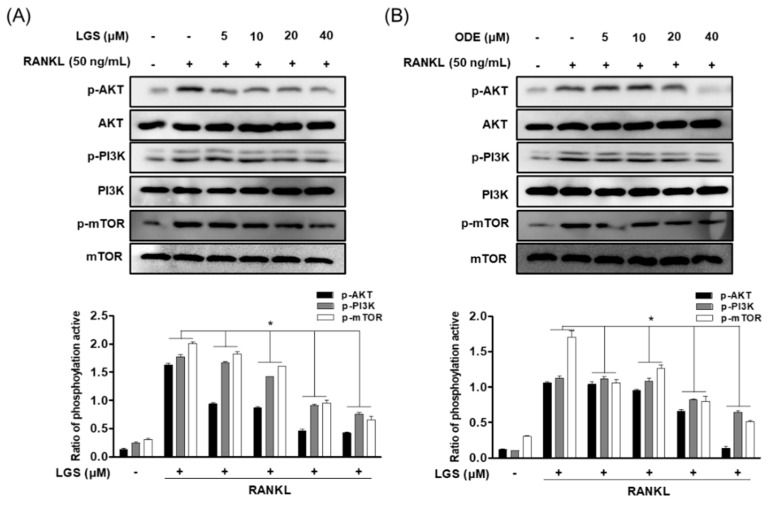
LGS and ODE inhibit the pathways of PI3K/Akt/mTOR in osteoclastogenesis induced by RANKL. RAW264.7 cells were treated with RANKL (50 ng/mL) at the indicated concentrations of LGS (**A**) and ODE (**B**) (5–40 μM) for 30 min. Thereafter, total protein was extracted from each group and evaluated by western blot. Relative protein expression levels were measured using ImageJ software. Mean value of three experiments ± S.D. was presented. * *p* < 0.05 versus the RANKL-treated group.

AKT/mTOR is associated with the major regulation of cellular energy homeostasis and induction of predation [22]. Therefore, the effects of LGS and ODE on the PI3K/Akt/mTOR signaling pathway upregulated by RANKL were investigated through western blotting. In the study results, the phosphorylation of PI3K/Akt/mTOR signaling was induced by RANKL treatment. The phosphorylation effect was inhibited in a concentration-dependent manner by treatment of each indicated concentration of LGS and ODE (Figure 8A,B). The inhibitory effects of RANKL on the phosphorylation of PI3K/Akt/mTOR signals, including the inhibitory effects of LGS and ODE on the phosphorylation of MAPKs shown above, are suggested to have an effect on various mechanisms in the process of inhibiting osteoclast differentiation by LGS and ODE.

## 3. Discussion

Natural medicines have high efficacy and few side effects, making them highly available for long-term use [23]. Drug therapy is often used to treat osteoporosis. In the case of currently developed treatments, there are drugs such as bisphosphonates or denosumab as inhibitors of bone resorption, but some patients treated with this drug experienced serious side effects such as uterine cancer or bone necrosis [24]. Therefore, finding a natural product-derived osteoporosis treatment without side effects seems to play an important role in the treatment of osteoporosis. Bone resorption due to excessive osteoclast formation and activity is closely related to osteoporosis, and thus inhibiting the differentiation and function of osteoclasts is an important therapeutic strategy for osteoporosis treatment [25]. In this study, the effects of the compounds ligustroside (LGS) and oleoside dimethylester (ODE) isolated from the *S. olbata* subsp. *dilatata* on RANKL-induced osteoclast differentiation and osteoclast function were evaluated.

RANKL is known to induce the expression of osteoclast-specific genes such as TRAP, DC-STAMP and Atp6v0d2 to induce differentiation and formation of mature osteoclasts, and upregulate the expression of important transcription factors for osteoclast differentiation such as NFATc1 [26]. In the results of this study, it was confirmed that LGS and ODE inhibited the actin ring formation, a structural feature of osteoclasts, and bone resorption, a function of osteoclasts during the differentiation and formation of osteoclasts by RANKL. According to previously reported studies, it is known that during RANKL-induced osteoclast formation, NF-κB and MAPK pathways (p38, JNK and ERK) are activated to induce downstream signaling and target gene expression [27,28]. The PI3K/Akt/mTOR signaling pathway is involved in a variety of cellular processes, including cell proliferation, differentiation, migration, and survival [29]. In addition, recent studies have demonstrated that in the process of osteoclast differentiation by RANKL, inhibitors of PI3K and mTOR down-regulate osteoclast formation and the level of TRAcP5 [30]. In this study, various signaling pathways were explored on the inhibitory effect of LGS and ODE on osteoclast differentiation, the effects of LGS and ODE were evaluated at the activation stage of the PI3K/Akt/mTOR signaling pathway, including NF-κB and MAPK by RANKL. As a result, LGS and ODE inhibited phosphorylation of NF-κB and translocation to the nucleus, and it effectively downregulated the phosphorylation of MAPKs proteins including ERK, p38 and JNK by RANKL. In addition, it was confirmed that the phosphorylation of the PI3K/Akt/mTOR signaling pathway by RANKL was also inhibited. Therefore, in the results of this study, it was found that LGS and ODE inhibit the phosphorylation activity of important cell signaling pathways such as NF-κB and MAPK activated by RANKL and PI3K/AKT/mTOR, which play an important role in the migration and proliferation of osteoclasts.

Bone disease therapeutics developed to date have various side effects caused by long-term administration, and due to this problem, development and research on new treatment methods and therapeutics for bone diseases have been proposed in recent years [31]. In the previously reported study results, the secoiridoid glucosides-based compound swertiamarin demonstrated the inhibitory effect of RANKL-induced inflammation and osteoclast differentiation, and suggested the possibility of a secoiridoid glucosides-based compound for the treatment of periodontal disease [32]. From these results, it is suggested that secoiridoid glucosides-based compounds can be effective regulators for inhibiting RANKL-induced osteoclast differentiation. In this study, it is also suggested that LGS and ODE, which are compounds of secoiridoid glucosides, can be used as treatments for bone diseases derived from natural products.

## 4. Materials and Methods

### 4.1. Chemicals and Reagents

Recombinant soluble RANKL ligand (RANKL) was purchased from PeproTech EC Ltd. (London, UK). Acid Phosphatase Assay kit, 3-(4,5-Dimethylthiazol-2-yl)-2,5-diphenyltetrazoliumbromide (MTT), Leukocyte acid phosphatase and 4-6-Diamidino-2-phenylindole (DAPI) was obtained from Sigma-Aldrich (Saint Louis, MO, USA). The minimum essential medium alpha (α-MEM) and fetal bovine serum (FBS) was purchased from Welgene Bioscience (Daegu, Korea). TRIZOL reagents were purchased from Thermo Fisher Scientific (Waltham, MA, USA), TOPscript™ RT DryMIX (dT18 plus) were obtained from Enzynomics (Daejeon, Korea). TB Green^®^ Premix Ex Taq™ II (Tli RNaseH Plus) was purchased from Takara (Tokyo, Japan). The primary antibodies for western blot assay phospho-JNK, JNK, phospho-p38, p38, phospho-IĸBα, IĸBα, phospho-PI3K, PI3K, phospho-Akt, Akt, mTOR and rabbit polyclonal antibodies were purchased from Cell Signaling Technology Inc. (Danvers, MA, USA). phospho-ERK, ERK, p65, phospho-mTOR, NFATc1 and c-Fos antibodies were obtained from Santa Cruz Biotechnology (Dallas, TX, USA). The enhanced chemilu-minescence (ECL) for western blotting detection system was obtained from Advansta Inc. (San Jose, CA, USA). The osteo-assay plate was purchased from Corning Inc. (corning, NY, USA). The real-time PCR primer of acid Phosphatase 5, tartrate Resistant (*acp5*), ATPase H+ Transporting V0 Subunit D2 (*atp6v0d2*) and dendritic cell-specific transmembrane protein (*dcstamp*) was purchased from Biomedic (Bucheon, Korea).

### 4.2. Isolation of LGS and ODE from the Twigs of S. oblata subsp. dilatata

The twigs of *S. oblata* subsp. *dilatata* were collected at Sungkyunkwan University campus, Suwon, Korea, in June 2014. The plant material was authenticated by one of the authors (K. H. Kim). A voucher specimen (SKKU-NPL 1404) was deposited in the herbarium of the School of Pharmacy, Sungkyunkwan University, Suwon, Republic of Korea. The dried twigs of *S. oblata* subsp. *dilatata* (6.9 kg) were extracted with 80% MeOH at room temperature three times. The extracts were filtered and concentrated under reduced vacuo to obtain a MeOH extract (450.0 g). Thereafter, the extract was suspended in H_2_O of hot, and resulting H_2_O layer was partitioned with *n*-hexane, CHCI_3_, EtOAc, and *n*-buOH to obtain resultant fractions of 15 g, 25 g, 48 g and 213 g, respectively. The EtOAc fraction (48.0 g) was separated by silica gel column eluting with CHCl_3_:MeOH (20:1, 15:1, 10:1, 6:1, 3:1 and 1:1) solvent conditions to give ten fractions (A–J). Subsequently, fraction F (1.5 g) was separated by using RP-C18 silica gel column eluting with 50% MeOH solvent condition and seven subfractions (F1–F7) were obtained. ODE (7 mg) was obtained from the subfraction F2 (274 mg) with 40% MeCN solvent condition by using semi-preparative HPLC. In the case of LGS (130 mg), it was isolated from the subfraction F4 (567 mg) with 30% MeCN solvent condition by semi-preparative HPLC. For their structural identification, nuclear magnetic resonance (NMR) spectra were measured from Bruker AVANCE III 700 NMR of Bruker (Karlsruhe, Germany). NMR solvent CD_3_OD was purchased from Sigma-Aldrich (Saint Louis, MO, USA). Chemical structures for compounds LGS and ODE were identified by comparison of the NMR data with those in the reference literature (Table 1) [33].

### 4.3. Cell Culture and Osteoclast Differentiation

RAW 264.7 murine macrophages were purchased from the American Type Culture Collection (ATCC, Rockville, MD, USA), and RAW 264.7 cells were cultured in Dulbecco’s modified Eagle’s medium (DMEM) and 10% fetal bovine serum (FBS), 100 U/mL Penicillin and streptomycin. For osteoclast differentiation, RAW 264.7 cells were into a 24-well plate at 5 × 10^3^ cells/well and cultured for 24 h. Then, the medium was changed with α-minimum essential medium (MEM) in each well using 10% FBS and 100 U/mL of penicillin and streptomycin. Incubate for 7 days after treatment with or without RANKL (50 ng/mL). After 7 days, osteoclasts are identified by Tartrate-resistant acid phosphatase (TRAP) staining or activity assay.

### 4.4. Cell Viability Assay

For the measurement of cell viability, RAW 264.7 cells were seeded in a 24-well plate at 5 × 10^3^ cells/well and cultured for 24 hr. After that, 5, 10, 20, 40 μM of LGS and ODE were treated for 7 days, respectively. Subsequently, 3-(4,5-dimethylthiazol-2-yl)-2,5-diphenyltetrazolium bromide (MTT) (5 mg/mL) was added to each well and incubated for 4 h. After 4 h, after removal of the MTT solvent, cells were lysed in DMSO (Dimethyl Sulfoxide), and absorbance was measured at 540 nm in a microplate reader (TECAN infinity pro 2000, Männedorf, Switzerland) to determine cell viability. Cell viability was expressed as a percentage of untreated control cells. Each group was analyzed individually 3 times.

### 4.5. Tartrate-Resistant Acid Phosphatase (TRAP) Activity and Staining

To evaluate the presence of Tartrate-resistant acid phosphatase (TRAP)-positive multinuclear cells and TRAP activity, the TRAP activity was confirmed using Sigma-Aldrich (Saint Louis, MO, USA) Acid Phosphatase Assay kit. RAW 264.7 cells were cultured in a 24-well plate at 5 × 10^3^ cells/well for 24 h and then treated with RANKL, LGS, and ODE for 7 days. Then, according to the manufacturer’s instructions, pick and dispense the supernatant from 96 wells, then dispense the substrate and let it react for 20 min in the incubator. Then add the stop solution, stop the reaction, and measure the absorbance at 405 nm. Quantification of TRAP activity was calculated according to the protocol of the Acid Phosphatase Assay Kit (Sigma-Aldrich, Saint Louis, MO, USA). For TRAP staining, cell staining was performed using an acidic phosphatase solution containing sodium tartrate according to the manufacturer’s protocol after fixation with 4% formaldehyde for 15 min and after cell fixation. Stained cells were examined under a microscope and digital pictures were taken.

### 4.6. Measurement of Actin Ring Formation

To detect the actin ring of osteoclasts, it was stained with Alexa Fluor 488 phalloidin. RAW264.7 cells were treated with RANKL (50 ng/mL) with LGS and ODE according to the indicated concentration to induce osteoclast formation. Specifically, RAW 264.7 cells were treated with LGS or ODE along with RANKL for 7 days, and fixed in 4% formaldehyde for 15 min to observe the formation of osteoclasts after 7 days. Thereafter, 0.5% of Triton X-100 was treated, stained with Alexa 488 and 4′,6-diamidino-2-phenylindole (DAPI), and photographed with a Nikon TE 200 fluorescence microscope (Tokyo, Japan). The quantification of actin ring was evaluated by counting the number of three or more multi-nuclei cells of each well (24 well plate), the counted of the only RANKL treatment group was percentage (%), and the only RANKL treatment group and the actin ring reduction of each group were compared.

### 4.7. Bone Resorption Assay

The area of bone resorption by osteoclasts was measured using osteo assay surface well (Corning, NY, USA). For bone resorption analysis, RAW264.7 cells were cultured in a 96-osteo assay surface well at a concentration of 2 × 10^3^ cell/well, and then simultaneously treated with 50 ng/mL of RANKL and LGS and ODE. After incubation for 7 days, the medium was removed and washed twice with phosphate-buffered saline (PBS), then treated with 5% sodium hypochlorite (Sigma-Aldrich, Saint Louis, MO, USA) for 5 min to remove cells, and the bone resorption area was photographed using an optical microscope. The bone resorption area of each well was measured by the Image J (National Institutes of Health, Massachusetts, MD, USA) program. Thereafter, the value was then percentage (%) of the only RANKL treatment group and compared with each of the resorbed area.

### 4.8. Real-Time Quantitative PCR

The gene expression levels of dcstamp, acp5 and atp6v0d2 were detected by Real-Time Quantitative PCR (RT-qPCR). Total RNA was extracted from the cell lysate using TRIZOL reagent, and then the concentration of mRNA was analyzed using Nano Drop. After that, cDNA was synthesized using TOPscript™ RT DryMIX (dT18 plus), and the real-time PCR reaction was run on a LightCycler 480 (Roche, Basel, Switzerland) instrument using TB Green^®^ Premix Ex Taq™ II (Tli RNaseH Plus). GAPDH was used as a housekeeping gene, and each was analyzed three times. The expression level of each gene was compared with GAPDH and averaged, and was calculated according to the following formula. 2^−ΔΔCT^, where ΔΔCT = (CTtarget − CTgapdh) at time x, −(CTtarget − CTgapdh) at time 0, time x represents any time point, and time 0 represents the 1 × expression of the gene in the untreated cells normalized to gapdh. The primers are presented in Table 2.

### 4.9. Western Blot Analysis

The RAW 264.7 cells were washed with PBS, lysed in a Radioimmunoprecipitation assay (RIPA) buffer containing protease and centrifuged at 13,000 rpm. Cell lysates were quantified by the Brad-ford method using the manufacturer’s kit (Bio-Rad, Hercules, CA, USA). Cell lysates were loaded and analyzed by SDS polyacrylamide gel electrophoresis and transferred to PVDF membrane (Bio-Rad, Hercules, CA, USA). To prevent non-specific binding, the membrane was blocked with 5% skim milk at room temperature, and then incubated primary antibody(phospho-p38, p38, phospho-ERK, ERK, phospho-JNK, JNK, Nfatc1, c-Fos, phospho-IĸBα, IĸBα, p65, phospho-PI3K, PI3K, phospho -Akt, Akt, phospho-mTOR and mTOR) at 4 °C overnight. After the membrane was washed, it was then incubated with secondary antibody (anti-rabbit IgG and anti-mouse IgG) for 2 h and detected with ECL (Advansta, CA, USA). The immunoreactive bands were analyzed by LAS 4000 (GE Healthcare Life Science, Chicago, IL, USA).

### 4.10. Statistical Analysis

The data were analyzed via means ± standard deviation (S.D.) of SigmaPlot software 12.1 (Systat Software Inc., San Jose, CA, USA). Student’s *t*-tests were used to compare two independent samples. *p* < 0.05 was considered statistically significant.

## 5. Conclusions

In this study, the effects of natural compounds LGS and ODE isolated from *S. olbata* subsp. *dilatata* were evaluated on RANKL-induced osteoclast differentiation and function, and various signaling pathways on osteoclast differentiation. In the results of this study, LGS and ODE effectively inhibited RANKL-induced osteoclast differentiation and function, and osteoclast-specific genes. Also, RANKL-induced translocation of NF-kB and phosphorylation of MAPK and PI3K/Akt/mTOR signals were inhibited. Therefore, LGS and ODE have the potential to inhibit osteoclast differentiation and are proposed as therapeutic agents to overcome bone disease caused by RANKL-mediated osteoclasts.

## Figures and Tables

**Table 1:** ^1^H (700 MHz) and ^13^C (175 MHz) NMR data of LGS and ODE (CD_3_OD).

Position	Ligustroside (LGS)		Oleoside Dimethyl Ester (ODE)	
	δ^1^H	δ^13^C	δ^1^H	δ^13^C
1	5.94 (1H, s)	95.3	5.95 (1H, s)	95.3
2	-	-	-	-
3	7.54 (1H, s)	155.3	7.55 (1H, s)	155.3
4	-	109.5	-	109.5
5	3.98 (1H, dd, *J* = 9.9, 4.2 Hz)	32.0	4.01 (1H, dd, *J* = 9.0, 4.2 Hz)	32.0
6	a: 2.72 (1H, dd, *J* = 14.1, 4.2 Hz) b: 2.44 (1H, dd, *J* = 14.1, 9.9 Hz)	41.4	a: 2.76 (1H, dd, *J* = 14.1, 4.2 Hz) b: 2.46 (1H, dd, *J* = 14.1, 9.0 Hz)	41.2
7	-	173.3	-	173.7
8	6.09 (1H, q, *J* = 6.6 Hz)	125.0	6.13 (1H, q, *J* = 6.3 Hz)	125.0
9	-	130.6	-	130.6
10	1.67 (3H, d, *J* = 6.6 Hz)	13.7	1.76 (3H, d, *J* = 6.3 Hz)	13.7
11	-	168.8	-	168.8
1′	a: 4.23 (1H, dt, *J* = 10.8, 6.9 Hz) b: 4.00 (1H, dd, *J* = 10.8, 6.9 Hz)	67.0	-	-
2′	2.83 (2H, t, *J* = 6.8 Hz)	35.3	-	-
3′	-	130.2	-	-
4′	7.08 (2H, d, *J* = 8.4 Hz)	131.1	-	-
5′	6.75 (2H, d, *J* = 8.4 Hz)	116.4	-	-
6′	-	157.2	-	-
7′	6.75 (2H, d, *J* = 8.4 Hz)	116.4	-	-
8′	7.08 (2H, d, *J* = 8.4 Hz)	131.1	-	-
1″	4.83 (1H, d, *J* = 7.8 Hz)	101.0	4.83 (1H, d, *J* = 7.8 Hz)	101.0
2″	3.33–4.82 (5H, m)	74.9	3.33–4.82 (5H, m)	74.9
3″		78.6	-	78.5
4″		71.6	-	71.6
5″		78.1	-	78.1
6″		62.9	-	62.8
COOCH_3_	3.69 (3H, s)	52.0	3.71 (3H, s)	52.0
COOMe	-	-	3.66 (3H, s)	52.3

**Table 2:** Primer sequences of real-time quantitative PCR analysis.

Target Gene	Sequence (5′-3′)	
*dcstamp*	Forward	TTTGCCGCTGTGGACTATCTGC
	Reverse	GCAGAATCATGGACGACTCCTTG
*acp5*	Forward	CGTCTCTGCACAGATTGCAT
	Reverse	GAGTTGCCACACAGCATCAC
*Atp6v0d2*	Forward	TGTGTCCCATTCTTGAGTTTGAGG
	Reverse	AGG GTCTCCCTGTCTTCTTTGCTT
*gapdh*	Forward	ACAGTCCATGCCATCACTGCC
	Reverse	GCCTGCTTCACCACCTTCTTG

## Data Availability

The data presented in this study are available in insert article.

## Sample Availability

Samples of the compounds are not available from the authors.
